# Characterization of Thermal Patterns Using Infrared Thermography and Thermolytic Responses of Cattle Reared in Three Different Systems during the Transition Period in the Eastern Amazon, Brazil

**DOI:** 10.3390/ani13172735

**Published:** 2023-08-28

**Authors:** Welligton Conceição da Silva, Jamile Andréa Rodrigues da Silva, Éder Bruno Rebelo da Silva, Antônio Vinicius Correa Barbosa, Carlos Eduardo Lima Sousa, Katarina Cardoso de Carvalho, Maria Roseane Pereira dos Santos, Kedson Alexandri Lobo Neves, Lucieta Guerreiro Martorano, Raimundo Nonato Colares Camargo Júnior, José de Brito Lourenço-Júnior

**Affiliations:** 1 Postgraduate Program in Animal Science (PPGCAN), Institute of Veterinary Medicine, Federal University of Para (UFPA), Federal Rural University of the Amazônia (UFRA), Brazilian Agricultural Research Corporation (EMBRAPA), Castanhal 68746-360, Brazil; eder.b.rebelo@gmail.com (É.B.R.d.S.); camargojunior@gmail.com (R.N.C.C.J.); joselourencojr@yahoo.com.br (J.d.B.L.-J.); 2 Institute of Animal Health and Production, Federal Rural University of the Amazônia (UFRA), Belem 66077-830, Brazil; jamileandrea@yahoo.com.br; 3 Cyberspace Institute, Federal Rural University of the Amazon, Belem 66077-830, Brazil; profvinibarbo@gmail.com; 4 Department of Veterinary Medicine, University Center of the Amazon (UNAMA), Santarem 68010-200, Brazil; cadu34.medvet@gmail.com (C.E.L.S.); katarinacc4@gmail.com (K.C.d.C.); 5 Institute of Engineering and Geosciences, Federal University of Western Pará (UFOPA), Santarem 68040-255, Brazil; roseanemaria022@gmail.com; 6 Institute of Animal Science, Federal University of Western Pará (UFOPA), Santarem 68040-255, Brazil; kedson_neves@hotmail.com; 7 Embrapa Eastern Amazon, Santarem 68010-180, Brazil; lucieta.martorano@embrapa.br

**Keywords:** heat, ambient climate, thermal stress, thermoregulation

## Abstract

**Simple Summary:**

This study aimed to characterize the thermal patterns of cattle raised in three different production systems during the transition period in the eastern Amazon, Brazil, using infrared thermography and by evaluating thermolytic responses. The evaluated systems were silvopastoral (with shade and no access to the bathing area), traditional (without shade and with no access to the bathing area), and integrated (with access to the shade and the bathing area). The thermographic images showed that the silvopastoral system, with shading, provided more favorable thermal conditions compared to the other systems. Physiological analyses revealed that the cattle maintained a rectal temperature within normal ranges in all systems. However, the respiratory rate was higher in the traditional system, indicating possible thermal stress in this environment. The thermal comfort indices indicated moderate stress conditions at times of greater solar intensity in all systems. In general, the results suggest that the silvopastoral system offers better thermal conditions for cattle, providing shade and reducing thermal stress. Thus, management practices aimed at animal welfare and productivity in production systems in the eastern Amazon region can be adopted by ranchers.

**Abstract:**

In the Lower Amazon mesoregion, there are basically three types of production systems: the traditional (without shade and no bathing area), the silvopastoral (with shade and no bathing area), and the integrated (with shade and bathing area). It is considered that the type of production system influences the thermal comfort and productivity of cattle, so this research aims to evaluate the influence of these three types of production systems on the thermoregulation of Nellore cattle. The experiment was carried out on a rural property for raising cattle, located in Mojuí dos Campos, Pará, Brazil, during the transition period (June/July). Thirty bovine males (not castrated, aged between 18 and 20 months, average weight of 250 ± 36 kg, body condition score of 3.5, clinically healthy) were randomly divided into three groups: Silvopastoral System—SS (*n* = 10), Traditional System—TS (*n* = 10), and Integrated System—IS (*n* = 10). Climate variables were collected (air temperature (AT °C), relative humidity (RH %), wind speed (WS, m/s), solar radiation (SR), black globe temperature (BGT °C), and physiological parameters, such as respiratory rate (RR) and rectal temperature (RT)) at 6 a.m., 12 p.m., 6 p.m., and 12 a.m. to determine the thermal comfort situation of the animals. Thermographic images of the environment and animals were captured in order to obtain the body surface temperature (BST) through infrared thermography. The Benezra Thermal Comfort Index (BTCI), Environmental Stress Index (ESI), Equivalent Temperature Index (ETI), and Iberian Heat Tolerance Index (Iberian HTI) were used. The results showed that the silvopastoral system, with shading by chestnut trees and an ample vegetative area, presented better thermal conditions, with an average of 28.98 °C, in comparison with the traditional system (35.93 °C) and the integrated one (34.11 °C). It was observed that the body surface temperature of cattle did not differ significantly between the anatomical regions of the body and the studied systems (*p* > 0.05). As for the respiratory rate, the traditional system registered higher values, with an average of 41 movements per minute, indicating possible thermal stress (*p* < 0.05). The thermal comfort indices revealed that all systems presented moderate stress conditions during times of higher solar intensity. It is concluded that the silvopastoral system proved to be more favorable for cattle, providing shade and reducing thermal stress, which may have a positive impact on animal welfare and productivity in this region.

## 1. Introduction

Cattle production in the eastern Amazon is an activity of great economic and social importance; with vast areas of natural pastures and rich biodiversity, the Amazon has significant potential for the development of cattle raising [1,2]. Thus, in the Amazon region, three models of ruminant rearing systems are mainly used: the traditional system, in which the animals do not have access to shade or water; the silvopastoral system, which provides shade for the animals, but without access to water for bathing; and the integrated system, which allows the animals to enjoy both shade and water for bathing.

The effects of these different production systems can lead to significant changes in the physiology and performance of these animals. Thus, it is possible to deduce that several weaknesses persist in the management of cattle. Furthermore, there is little information about the influences that these different breeding environments can have on beef cattle produced in the region. Thus, it can be inferred that there are no studies about the occasional influences on beef cattle resulting from these breeding environments widespread in the region, which justifies this study.

In this context, some indices help in determining the resulting influences of these production systems, such as the Temperature and Humidity Index (THI), the Black Globe Temperature and Humidity Index (BGTI), the Benezra Thermal Comfort Index (BTCI), the Environmental Stress Index (ESI), the Temperature Equivalent Index (ETI), and the Iberian Heat Tolerance Index (Iberian HTI), calculated according to meteorological variables and physiological parameters [3,4,5,6,7]. Thus, in livestock, infrared thermography has proven to be a valuable tool for monitoring animal health and well-being, allowing for the detection of thermal anomalies, the identification of injuries, the evaluation of the thermal performance of installations, and support for the early diagnosis of health, thus providing a more efficient and precise approach to the management and care of herds [8,9,10,11].

Thus, infrared thermography (ITR) helps in the determination of thermal patterns, being a passive, remote, and non-invasive thermal visualization technology that helps in the determination of high temperature zones through images obtained, which directly demonstrate the distribution temperature on the surface of the animals and in the studied production systems [9,12,13].

Cattle are animals sensitive to temperature variations, and when exposed to conditions of excessive heat, they can face serious health and well-being problems. During periods of high temperatures, cattle have difficulties in dissipating body heat, which leads to an increase in respiratory rate and sweating as thermoregulation mechanisms; this suffering is directly influenced by the rearing system [14].

Furthermore, the increase in body temperature is directly related to the increase in physiological parameters, such as respiratory rate, heart rate, and panting score; all this can be used as a sign of thermal stress [15,16]. According to McDowell et al. [17], an increase in rectal temperature of 1 °C is enough to reduce the productivity and performance of different animal species, which results in difficulties in weight gain and adaptive processes.

In this way, the present study presented relevant data about the raising of cattle in systems that include shaded areas and access to water for bathing. This information offers valuable insights for the adoption of appropriate management practices in the production of beef cattle in the eastern Amazon region. Therefore, the objective of this study was to characterize the thermal patterns using infrared thermography and thermolytic responses of cattle reared in three different systems during the transition period in the eastern Amazon, Brazil.

## 2. Materials and Methods

### 2.1. Ethical Aspects

This study was submitted to the Committee for Ethics in the Use of Animals (CEUA) and obtained Approved status, under protocol CEUA-UNAMA 0001-87/2023, in May 2023.

### 2.2. Location 

**Figure 1:**
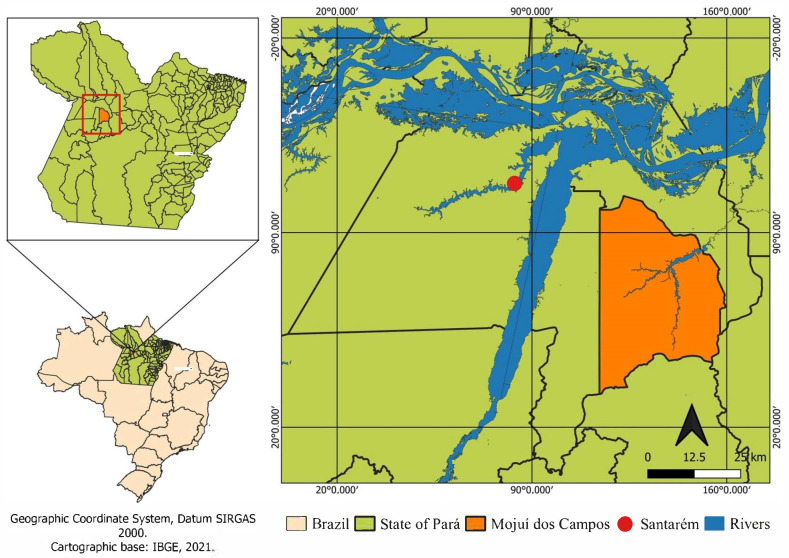
Location map of the study area.

This study was carried out on a rural property for raising cattle, located in Mojuí dos Campos, Pará, Brazil (Figure 1), in the transition period of the year (rainier to less rainy—June/July).

The climate of the mesoregion is hot and humid (Am4); it is characterized by total rainfall lower than 60 mm in the least rainy month and by annual rainfall between 1900 and 2100 mm. It is also characterized by an annual average air temperature of 25.6 °C and relative humidity ranging from 84 to 86% [18]. The rainiest quarter occurs between the months of February and April, and the least rainy quarter occurs between the months of August and October [19]. The forage plant *Brachiaria humidicola* was implanted in the systems.

### 2.3. Experimental Animals, Management, and Characterization of the Production System

**Figure 2:**
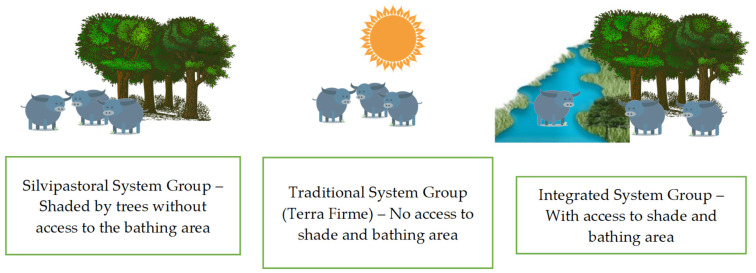
Infographic of the experimental design by evaluated group.

Thirty Nellore bovines (male, not castrated, with similar coloring, aged between 18 and 20 months, average weight of 250 ± 36 kg, body condition score of 3.5 (scale from 1 to 5), clinically healthy) were randomly divided into three groups: Silvopastoral System—SS (*n* = 10), Traditional System—TS (*n* = 10), and Integrated System—IS (*n* = 10) (Figure 2). The SS group remained in a paddock of the same size, with around 20% shade from chestnut trees (*Bertholletia excelsa*), with access to drinking water and mineral salt *ad libitum*. The TS group was led to a paddock without shade from trees, with a *Brachiaria humidicola* pasture, and access to drinking water and mineral salt ad libitum. The IS group remained in a paddock of the same size and pasture, but with access to 20% shade from Brazil nut trees (*Bertholletia excelsa*) and access to water for drinking and bathing, as well as mineral salt, ad libitum. The total experimental area was 10.2 ha of *Brachiaria humidicola*, divided into six paddocks of 1.7 ha, with two per treatment.

The period of adaptation of the animals to the handling was seven consecutive days, where the animals were taken to the chute so that data of the physiological variables could be collected during the experiment. The production systems used are characterized as follows:

I. Traditional System—no shade and no access to the bathing area. In this system, the animals were subjected to pasture without the presence of trees or other elements that could provide shade; they also did not have access to the bathing area.
II. Silvopastoral System—with shade and no access to the bathing area. In this system, the animals were subjected to pasture with the presence of trees and other elements that could provide shade; they also did not have access to the bathing area.
III. Integrated System—with access to shade and bathing area. In this system, the animals were subjected to pasture with trees and other elements that can provide shade, as well as access to the bathing area.

### 2.4. Meteorological Variables

**Figure 3:**
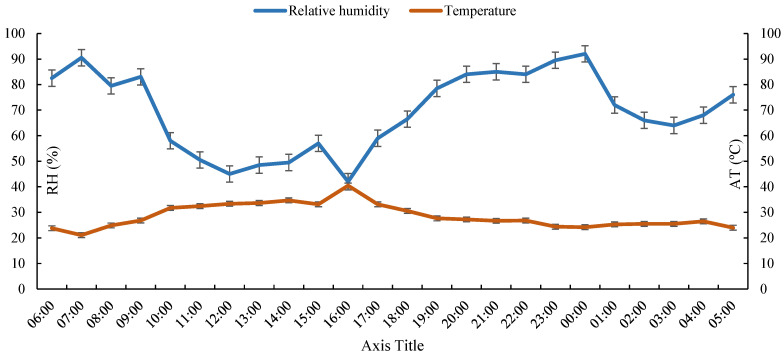
Average agrometeorological data during the period of the experiment.

The climatic variables evaluated were air temperature (AT °C), relative air humidity (RH %), wind speed (WS, m s^−1^), dew point temperature (DPT °C), wet bulb temperature (WBT °C), and black globe temperature (BGT °C) obtained using a conventional station. The microclimate conditions, specifically temperature and humidity during this study, are shown in Figure 3.

### 2.5. Physiological Variables

All physiological variables were collected at 6:00 a.m., 12:00 p.m., 6:00 p.m., and 12:00 a.m. during the transition period (June and July) during two days of performance in the months of June and July. On the days of collection, the animals were taken to the management corral, remaining on hold for 30 min before the start of activities to avoid the influence of management on the physiological variables. For this, the animals were managed to the corral on foot and contained in batch type trunks in environments protected from direct sunlight and rain. The animals were handled in batches of ten animals to the corral, avoiding a fixed order of animal entry, and preventing animal data from being influenced by advancing time, within the stipulated time window. 

### 2.6. Respiratory Rate (RR)

The RR was obtained by inspecting and counting the thoracic-abdominal movements for one minute [20] with the help of a digital stopwatch. Data were collected in the transition period (June/July) during two days of the experiment at 6:00 a.m., 12:00 p.m., 6:00 p.m., and 12:00 a.m. This assessment was carried out by a single, previously trained, observer.

### 2.7. Rectal Temperature (RT)

The RT was obtained using a veterinary clinical thermometer (Model-5198.10, In-coterm^®^, São Paulo, Brazil), with a maximum scale of up to 44 °C inserted transrectally into the animals, with the results expressed in degrees Celsius, as per described by Dirksen et al. [21]. This evaluation was carried out by a single observer, during the transition period (June/July) during two days of the experimental period, at 6:00 a.m., 12:00 p.m., 6:00 p.m., and 12:00 a.m.

### 2.8. Infrared Thermography

The diagnosis of the thermal patterns of the targets in the environment of the three production systems was carried out using infrared thermography, and this collection was carried out on 1 July 2023 in an area that totals 1.7 ha per production system. Thus, thermographic image captures were carried out between 12:00 and 15:00, a period with an intense effect of solar radiation on the targets diagnosed in the field research. Thermographic images were acquired on the right side of the animals to reflect the real fluctuation of the IRT, thus preventing interference from ruminal movements. 

We monitored the targets by taking images with a near-infrared thermograph (FLIR T650sc), considering an emissivity of 0.95 because it is an open system composed of different materials. Thermograms were analyzed using the Flir Tools program, 6.3 v [22], with the Rainbow HC palette chosen.

The images were acquired from four areas: the head region, the armpit, the flank, and the rump, according to the description of thermal windows described in Mota-Rojas et al. [10]. 

The camera had high precision with a fixed 25 mm lens, a temperature range from −40 to 150 °C, and thermal sensitivity of 50 mK (>0.05 °C at an ambient temperature of 30 °C). The spectral range ranged from 0.7 to 100 μm, but the imaged targets presented a response between 0.7 and 3.0 μm and an optical resolution of 640 × 480 pixels. 

### 2.9. Body Surface Temperature (BST)

**Figure 4:**
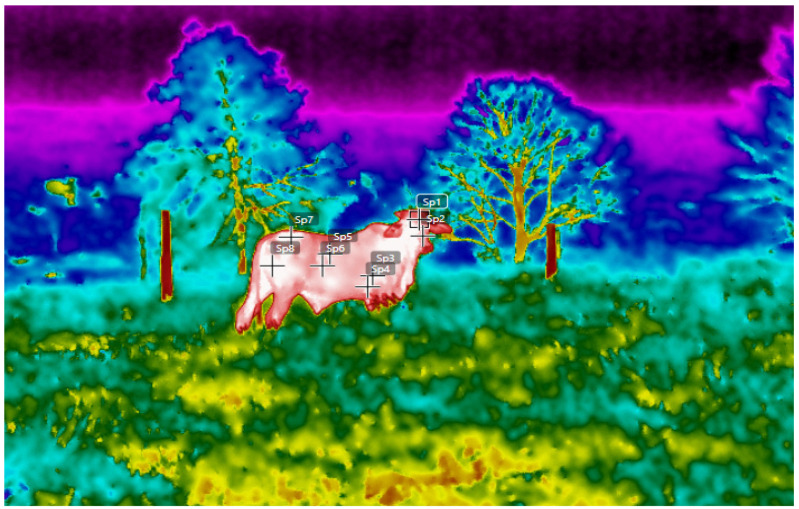
Points imaged using infrared thermography. Anatomical region—SP1 and SP2—head; SP3 and SP4—armpit; SP5 and SP6—flank; and SP7 and SP8—rump.

The BST was obtained with the aid of an infrared thermometer using the scientific thermographic camera in the transition period (June/July); it was specifically carried out on 1 July 2023, from 12:00 to 15:00, in the anatomical regions of the head, the armpit, the flank, and the rump, with ten points being imaged (Figure 4). The thermograms of the animals were acquired in the field, while in the pastures, and with the animal in a standing position. In the systems, thermograms were acquired at an approximate orthogonal distance of 5 m, outside the flying distance of the bull [23], so that the animals can remain calm and in their natural behavior.

### 2.10. Heat Storage Calculation

The heat storage calculation, in kg of kelvin^−1^ h^−1^, was obtained using the formula [24]:

ΔRT = (3600 × Harm × A)/(Mv × Hb)

where ΔRT—difference in RT; Harm—stored heat (W·m^−2^); A—animal’s surface (m^2^) calculated by the equation: A = 0.13·M^0.556v^; Mv–body mass (kg); and Hb—animal’s specific heat (3400 kJ kg^−1^ K^−1^).

### 2.11. Index to Assess Thermal Comfort

#### 2.11.1. Temperature and Humidity Index (THI)

During the experiment, readings of environmental variables were collected simultaneously with measurements of physiological variables throughout the day. From the values recorded for the environmental conditions, the Temperature and Humidity Index (THI), proposed by Thom [25], was calculated using the formula:

THI = DBT + 0.36 × DPT + 41.5

where DBT = Dry bulb temperature (°C) and DPT = Dew point temperature (°C).

THI = temperature and humidity index, indicating <72 (green color); >72 to 78 (yellow color); and 79 to 88 (orange color) [25]. 

#### 2.11.2. Black Globe Temperature and Humidity Index (BGHI)

From obtaining these variables, the index of the temperature of the black globe and humidity (BGHI) will be calculated, according to what was proposed by Buffington et al. [26], using the equation:

BGHI = BGT + 0.36 (DPT) + 41.5

where BGHI = Black Globe Temperature and Humidity Index, BGT = black globe temperature (°C), and DPT = dew point temperature (°C).

BGHI = black globe temperature and humidity index, indicating >72 comfort situation (green color) and 74 to 78 warning (yellow color) [26].

#### 2.11.3. Benezra Comfort Index (BTCI)

The Benezra Thermal Comfort Index (BTCI) [27] was calculated by the formula:

BCI = RT/38.33 + RR/23

where RT = rectal temperature (°C) and RR is the respiratory rate.

A BTCI with values greater than 2.0 indicates difficulty in the degree of adaptation of the animals to the environment. Values below 2.0 indicate greater ease of adaptation [27].

#### 2.11.4. Environmental Stress Index (ESI)

The Environmental Stress Index (ESI) was calculated using the formula [28]:

ESI = 0.63DBT − 0.03RH + 0.002SR + 0.0054(DBT × RH) − 0.073(0.1 × SR)^−1^

where DBT = dry bulb temperature, in °C; RH = relative humidity, in %; and SR = solar radiation, in W·m^−2^.

ESI = environmental stress index, indicating <25 low risk (green color) and ≤25 to ≤33 moderate to high risk (yellow color) [28]. 

The Equivalent Temperature Index (ETI) is a metric developed by Baeta et al. [29], and it takes into account temperature, humidity, and air velocity, being especially applicable for semi-housed animals. This index was calculated using the following formula:

ETI = 29.83628 − 0.11519 × AT + 0.00059 × RH − 0.30525 × WS

where ETI = Equivalent Temperature Index; AT = Air temperature in °C (dry bulb); RH = relative air humidity, in %; and WS = wind speed, in m/s.

ETI = equivalent temperature index, indicating no problems from 18 to <27 (green color) and caution ≥27 to ≥32 (yellow color) [29].

#### 2.11.5. Iberian Heat Tolerance Index (Iberian HTI)

The Iberian Heat Tolerance Index (Iberian HTI) [30] was calculated using the formula:

HTI = 100 − 18(RT − 38.33)

where HTI = Heat Tolerance Index and RT = rectal temperature.

An Iberian HTI index close to 100 indicates greater animal comfort [30].

### 2.12. Data Analysis

The relationships between the thermal comfort indices and the physiological stress indices were investigated. The climatological data were subjected to descriptive statistics analysis. Statistical analyses were performed in Software R version 4.3.1 (RStudio 2023.03.0 Build 386) using parametric ANOVA with Tukey’s post hoc test and non-parametric with chi-square test for equal expected proportions when comparing the times between themselves. All analyses considered a significance level of 5%. Statistical analyses for the TR and FR variables were performed considering repeated measurements over time and with interaction between the rearing systems and schedules, with a parametric analysis being performed after meeting the assumptions of normality of the residues by the Shapiro–Wilk test (*p*-value > 0.05) and the assumption of equality of variances by Mauchly’s sphericity test at 5% significance. After ANOVA, the student’s *t*-test was performed.

## 3. Results

**Figure 5:**
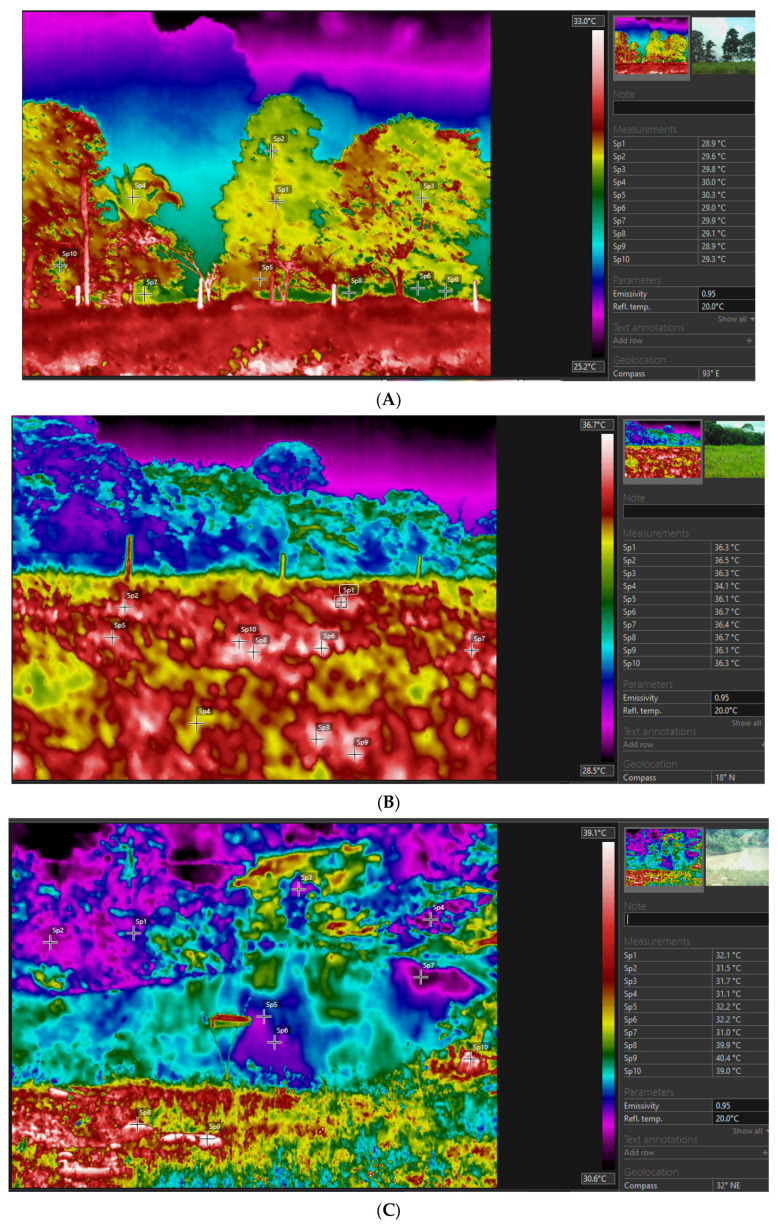
Thermographic images of the silvopastoral (**A**), traditional (**B**), and integrated (**C**) systems of cattle raising observed between 13:00 and 15:00 during the experimental period in the eastern Amazon. Description of the systems: Silvopastoral—with shade and no access to the bathing area. In this system, the animals were submitted to pasture with the presence of trees and other elements that could provide shade; they did not have access to the bathing area. Traditional—no shade and no access to the bathing area. In this system, the animals were subjected to grazing without the presence of trees or other elements that can provide shade; they did not have access to the bathing area. Integrated—with access to shade and bathing area. In this system, the animals were subjected to pasture with trees and other elements that can provide shade, as well as access to the bathing area.

In Figure 5, thermographic images can be seen in the three different production systems, in which the silvopastoral system (Figure 5A) expresses the thermal conditions in the trees in the URT containing an area shaded by chestnut trees (*Bertholletia excelsa*), with a large vegetative area, which favors the provision of shade and reduces the temperature when compared with the thermal images of the traditional system (Figure 5B) and the integrated system (Figure 5C). In the traditional system, it is noted that the temperatures were higher than the other systems because there is no presence of trees. The integrated system with the supply of water for bathing combined with shading with chestnut trees (*Bertholletia excelsa*) presented values higher than the silvopastoral system and lower than the traditional one.

In Table 1, the values of the thermal conditions in each animal production system are noted, and a statistical difference between the silvopastoral system for the traditional and integrated (*p* < 0.05) systems is observed, with no difference between the traditional and integrated (*p* > 0.05).

Regarding the body surface temperature (BST), it was noted that the cattle did not show any difference between the anatomical regions of the body and the systems studied (*p* > 0.05) (Table 2).

**Figure 6:**
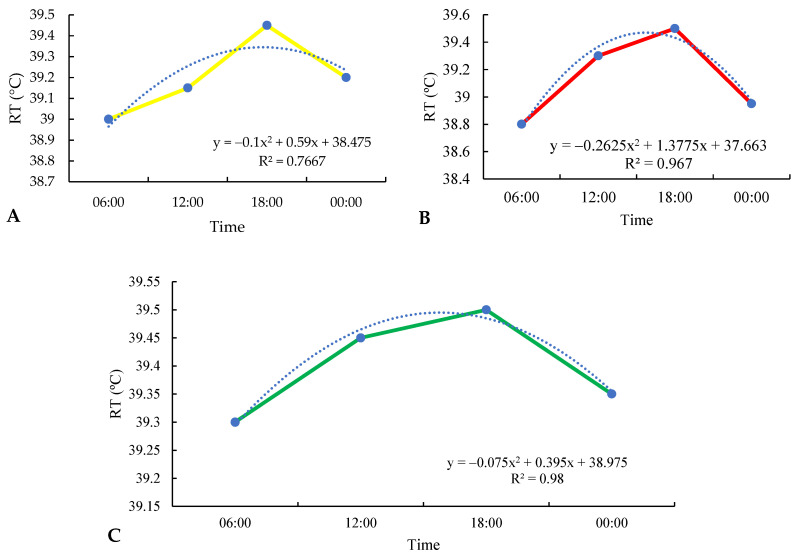
Rectal temperature (RT) in the silvopastoral (**A**), traditional (**B**), and integrated (**C**) systems of cattle observed at 6:00 a.m., 12:00 p.m., 6:00 p.m., and 12:00 a.m. during the experimental period in the eastern Amazon. Description of the systems: Silvopastoral—with shade and no access to the bathing area. In this system, the animals were submitted to pasture with the presence of trees and other elements that could provide shade; they did not have access to the bathing area. Traditional—no shade and no access to the bathing area. In this system, the animals were subjected to pasture without the presence of trees or other elements that could provide shade; they did not have access to the bathing area. Integrated—with access to shade and bathing area. In this system, the animals were subjected to pasture with trees and other elements that can provide shade, as well as access to the bathing area. Dotted line indicates trend line.

In the regression analysis of rectal temperature as a function of collection times (06:00, 12:00, 18:00, and 00:00), it was possible to observe that in the silvopastoral, traditional, and integrated systems, there was a higher index of this parameter at 18:00, reaching values of 39.45 °C, 39.5 °C, and 39.5 °C, respectively (Figure 6A–C). Note that there was a gradual increase in the mean TR at 6 a.m., 12 a.m., and 6 p.m., with an exponential reduction at 12 a.m. in all evaluated systems. There was no statistical difference between times per treatment (*p* > 0.05). However, there was a difference between the hours (*p* < 0.05). There was no difference in the treatment and time interaction (*p* > 0.05). There was a difference between 00:00 and 18:00 within the silvopastoral treatment and between 6:00 and 18:00 in the integrated system. In the grouping of times, a difference was observed between all times, except between 00:00 and 12:00. The TR is considered normal for cattle when it is in the range of 38 to 39.5 °C [20]. In this study, cattle reared in silvopastoral, traditional, and integrated systems were within normal limits, with no thermal stress.

**Figure 7:**
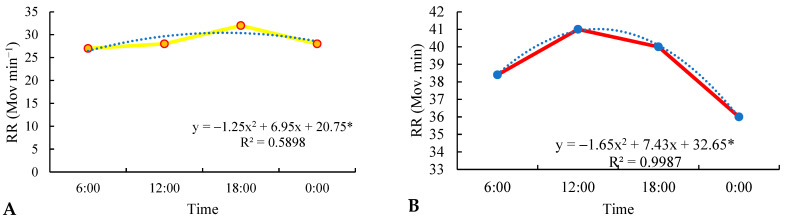

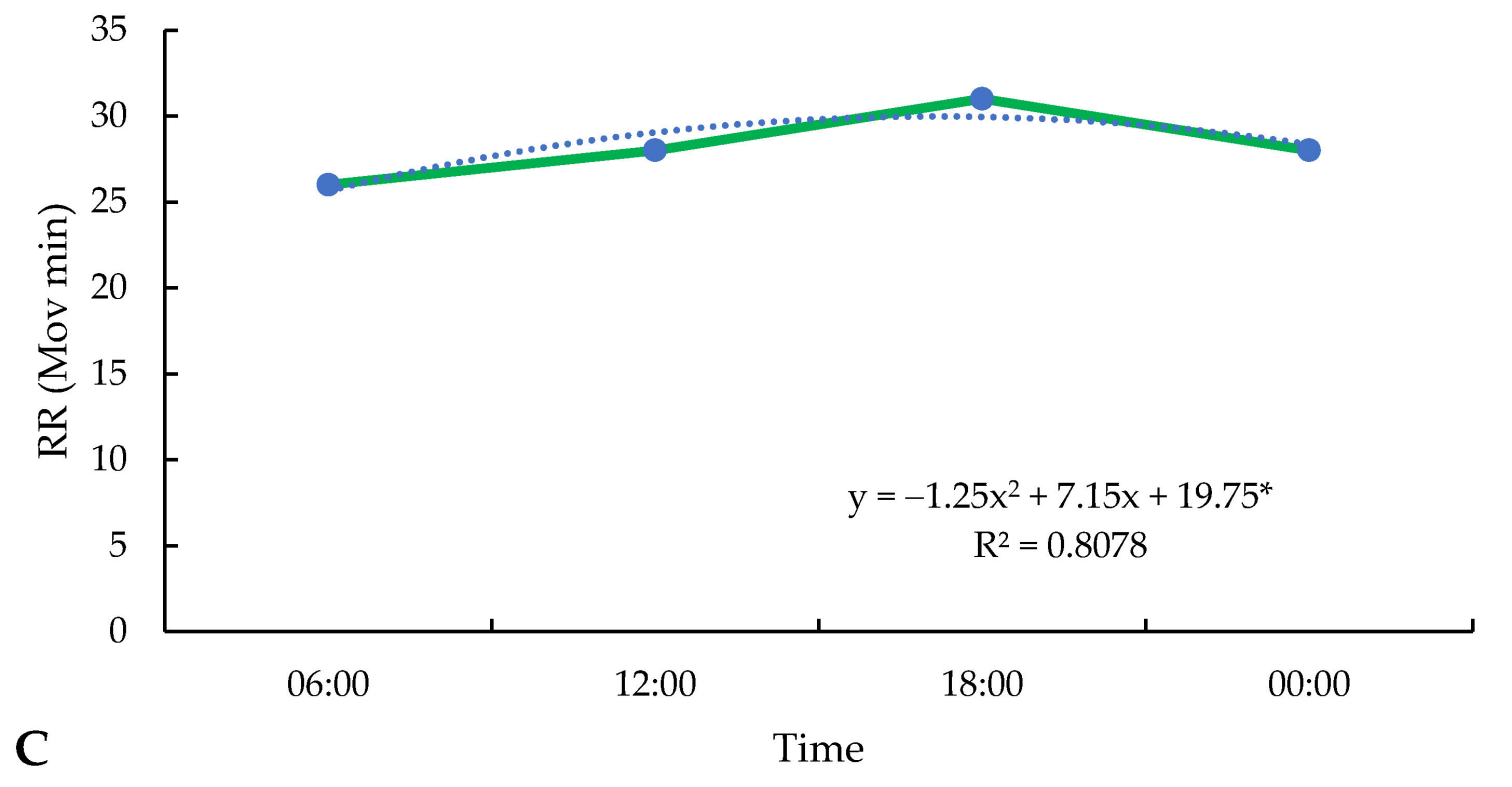
Respiratory rate (RR) in the silvopastoral (**A**), traditional (**B**), and integrated (**C**) systems of cattle observed at 6:00 a.m., 12:00 p.m., 6:00 p.m., and 12:00 a.m. during the experimental period in the eastern Amazon. * *p* < 0.05. Description of systems: Silvopastoral—with shade and no access to the bathing area. In this system, the animals were submitted to pasture with the presence of trees and other elements that could provide shade; they did not have access to the bathing area. Traditional—no shade and no access to the bathing area. In this system, the animals were subjected to pasture without the presence of trees or other elements that can provide shade; they did not have access to the bathing area. Integrated—with access to shade and bathing area. In this system, the animals were subjected to pasture with trees and other elements that can provide shade, as well as access to the bathing area. Dotted line indicates trend line.

The regression analysis in relation to the respiratory rate (RR) in the three rearing systems revealed a quadratic effect in relation to the studied time, as observed in Figure 7A–C. When deriving the equation, it was identified that the maximum RR reached 32, 41, and 31 mov min^−1^ in the silvopastoral, traditional, and integrated systems at 6:00 p.m., 12:00 a.m., and 6:00 p.m., respectively (*p* < 0.05) (Figure 7A–C). Added to this, throughout the day, there was a gradual increase in RR in the three rearing systems, with the highest increase observed in animals raised in the traditional system; that is, without the presence of shade, especially at 12:00 a.m., with an average RR of 41 mov min^−1^. This upward trend of RR throughout the morning and early afternoon indicates a possible physiological response of the study subjects to environmental changes or daily activities. The RR was within the reference values described by Feitosa [20] (within the range of 10 to 30 movements per minute for the silvopastoral system at 6:00 a.m., 12:00 a.m., and 00:00 p.m., with thermal stress at 6:00 p.m. (32 mov min^−1^)), with the same dynamics observed for the animals in the integrated system. However, for the traditional system, stress was noted at all times (6:00 a.m., 12:00 p.m., 6:00 p.m., and 12:00 a.m.), with RR greater than 30 mov min^−1^ [20]. There was a difference for treatment, time, and treatment and time interaction (*p* < 0.05). There was a difference between the silvopastoral and traditional systems and between the traditional and integrated systems (*p* < 0.05); however, there was no difference between the traditional and integrated systems (*p* > 0.05).

**Figure 8:**
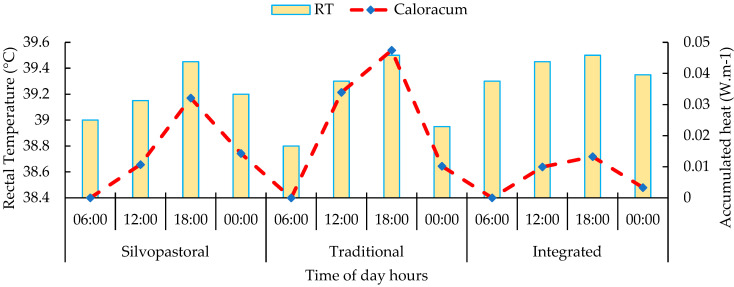
Thermal balance of the interaction between RT and heat accumulation in silvopastoral, traditional, and integrated systems at 06:00, 12:00, 18:00, and 00:00 during the experiment in the eastern Amazon. Description of the systems: Silvopastoral—with shade and no access to the bathing area. In this system, the animals were submitted to pasture with the presence of trees and other elements that could provide shade; they did not have access to the bathing area. Traditional—no shade and no access to the bathing area. In this system, the animals were subjected to pasture without the presence of trees or other elements that can provide shade; they did not have access to the bathing area. Integrated—with access to shade and bathing area. In this system, the animals were subjected to pasture with trees and other elements that can provide shade, as well as access to the bathing area.

There was a greater accumulation of heat during the period of the experiment at 12:00 and 18:00 in the traditional and integrated system and at 18:00 and 00:00 in the silvopastoral system, all associated with the highest rectal temperature index (Figure 8).

In the THI assessment, it was possible to observe the absence of thermal stress at 7:00 a.m. (<72). On the other hand, lower rates were identified at 6:00 a.m., 8:00 a.m., and 9:00 a.m. and after 7:00 a.m., characterizing mild stress (>72 to 78). Moderate stress was evidenced at times of greater solar intensity (from 10:00 a.m. to 6:00 p.m.). The BGHI demonstrated comfort indices in the early hours (6:00 a.m., 7:00 a.m., and 8:00 a.m.) of the day and from 11:00 p.m. to 6:00 a.m.. However, moderate stress was presented between 9:00 a.m. and 10:00 a.m. and from 1:00 p.m. to 10:00 p.m. (Table 3). The environmental stress index (ESI) pointed to a moderate risk at 10:00 p.m., corroborating data from the BGHI, while the other times showed a low risk of stress. The equivalent temperature index (ETI) signaled a state of caution (≥27 to ≥32) at 7 a.m., with the other times showing no stress (18 to <27).

The Iberian HTI showed no difference between the studied production systems (*p* > 0.05). The BTCI had a higher index in the traditional system (*p* < 0.05) when compared with the silvopastoral and integrated systems. For the times of day, no difference was observed for either the Iberian HTI or the BTCI. However, the morning shift (87.34) was the most comfortable for the welfare of the cattle, as it had an Iberian HTI index close to 100. In the case of the BTCI, all values were greater than 2.0, signaling difficulty in the degree of adaptability of the animals to the environment (Table 4).

## 4. Discussion

The meteorological variables showed higher rates of AT at the hottest times of the day, specifically from 11:00 a.m. to 5:00 p.m., this being the period of highest solar intensity and the lowest RH of the air. Therefore, these are characterized as stress factors due to high temperatures, high humidity, and exposure to intense solar radiation at the times mentioned above [26,33,34,35,36]. In a study carried out by Hooper et al. [37], it was observed that air and surface temperatures are higher when there is a decrease in RH during the afternoon compared to the morning, influencing the physiological characteristics of Nellore animals.

The use of infrared thermography proved to be an efficient tool to evaluate and compare the three different cattle production systems in the eastern Amazon. The captured thermographic images clearly revealed the differences in the thermal conditions of the silvopastoral, traditional, and integrated systems. The study by Cândido et al. [38] sought to evaluate the silvopastoral system through infrared thermography, thus reinforcing the effectiveness of the technique.

Figure 6A, which represents the silvopastoral system, shows areas shaded by trees, mainly by the presence of chestnut trees (*Bertholletia excelsa*), which provide a cooler and more comfortable environment for the animals. This large vegetative area acts as a barrier against direct solar radiation, reducing the absorption of heat by the animals and, consequently, decreasing the local temperature [39,40,41], as shaded areas can reduce the heat load by up to 30% [42].

In contrast, Figure 6B, which depicts the traditional system without tree shading, shows higher temperatures in relation to the silvopastoral system. Without the protection of the trees, the animals are exposed to direct solar radiation and the heating of the soil, resulting in a significant increase in body temperature. Figure 6C, representing the integrated system with shading provided by the trees and the supply of water for bathing, shows intermediate temperatures between the silvopastoral and traditional systems. Tree shading contributes to thermal relief [23,43,44,45,46,47,48,49,50], while the availability of bathing water allows animals to regulate their body temperature through water evaporation, providing a more pleasant environment.

Another justification for the integrated system exhibiting higher temperatures compared to the silvopastoral system is due to the lower leaf density and wider spacing within the tree canopies. The sparser foliage in the integrated system allows for greater penetration of direct solar radiation through the canopy, resulting in increased heating of the underlying environment. In contrast, the silvopastoral system, characterized by more densely distributed leaves within the tree canopies, tends to filter a significant portion of direct solar radiation, contributing to a milder temperature within the system.

On the other hand, the absence of trees in the traditional system can lead to greater exposure of animals to solar heat, resulting in higher temperatures. Studies show that direct solar radiation can considerably increase the temperature of the soil surface and, consequently, the body temperature of animals, providing indices of thermal stress [51].

The BST showed no difference (*p* > 0.05) between the systems and for the anatomical regions studied; this can be explained by the combination of environmental, management, and adaptive factors that may have leveled the thermal responses of the animals in study.

The analysis of rectal temperature regression at different times (6:00 a.m., 12:00 a.m., 6:00 p.m., and 12:00 a.m.) in silvopastoral, traditional, and integrated systems is important to understand how animals respond to thermal variations throughout the day and how these production systems can influence the heat stress in cattle. Thus, the results show that there was a gradual increase in RT throughout the day, reaching its maximum peak at 6:00 p.m. in all evaluated systems. This increase in RT is related to the increase in ambient temperature during the day, especially in the hours of greater solar intensity. Direct solar radiation and ambient temperature can lead to an increase in thermal stress in animals, leading to an increase in RT [52,53].

However, despite the increase in RT throughout the day, the values observed in the silvopastoral, traditional, and integrated systems, with temperatures of 39.45 °C, 39.5 °C, and 39.5 °C, respectively, still remained within the range considered normal for cattle (38 to 39.5 °C), indicating the absence of thermal stress in the animals.

These results are in line with the scientific literature that highlights the importance of shading in silvopastoral systems to reduce heat stress in cattle. The presence of trees in silvopastoral systems provides shading, which reduces direct exposure to solar radiation and helps to reduce the accumulation of heat in the environment [51,54,55,56,57,58,59]. In addition, the presence of bathing areas, as in the integrated system, can also contribute to heat dissipation by the animals.

The regression analysis of RR in the three cattle breeding systems revealed a quadratic effect in relation to collection times, which indicates a significant variation of this parameter throughout the day. This variation may be related to environmental changes, such as fluctuations in temperature and solar radiation, which directly affect thermal stress in animals. The traditional system showed higher RR values at all times, indicating that the animals in this system are under greater thermal stress, probably due to the lack of shading and adequate spaces for bathing. The silvopastoral and integrated systems showed RR within the reference values, with heat stress evident only at 6 p.m. [20].

The physiological response observed in animals reared in the traditional system can be attributed to direct exposure to solar radiation and the lack of mechanisms to dissipate excessive heat. When cattle face high temperatures, their thermoregulatory system kicks in, increasing the RR to facilitate heat dissipation through breathing. This increase in RR is a way for animals to lose heat by evaporating water from the respiratory surface, but this physiological response can also indicate discomfort and heat stress [41,60].

On the other hand, the silvopastoral and integrated systems provide shaded areas and spaces for bathing, allowing cattle to protect themselves from excessive heat and maintain a more balanced body temperature [61,62]. The shade provided by the trees in the silvopastoral system and the availability of water for bathing in the integrated system help to reduce the ambient temperature and, consequently, the need to dissipate heat through respiratory rate. This results in lower RR values in these systems, keeping cattle within normal heat stress limits [63,64].

The RR regression analysis in the three cattle breeding systems revealed a quadratic effect in relation to collection times. This means that the RR showed significant variation throughout the day, with a curvilinear pattern, reaching maximum values at 6:00 p.m., 12:00 p.m., and 6:00 p.m. in the silvopastoral, traditional, and integrated systems, respectively. This variation indicates that cattle are physiologically responding to environmental changes, especially in relation to temperature and heat stress. Thus, in situations of thermal stress, sweating and peripheral vasodilation initially occur, resulting in a reduction in blood pressure, compensated by an increase in heart and respiratory rate [65,66,67]. These changes generate a greater demand for energy [68] and can lead to reduced productivity [69]; as a result, the need to maintain homeothermy leads to changes in basic physiological patterns, such as respiratory rate and rectal temperature. Thus, the monitoring of these variables becomes useful to assess the thermal balance in animals [70].

Heat stress in cattle can lead to an increase in RR as an adaptive response to reduce body heat. RR is an important indicator of thermal well-being in animals, and its elevation can indicate discomfort and thermal stress [71,72,73,74,75]. Hahn et al. [76] reported an increase in breathing rate of about 4.3 breaths per minute above the basal rate, i.e., 60 breaths per minute, for every degree Celsius above the threshold temperature of 21.3 °C.

The RR regression analysis in cattle-raising systems showed the importance of shading and providing bathing areas as effective strategies to reduce heat stress in animals. These thermal comfort measures can minimize the physiological responses of cattle to heat, promoting greater well-being and better production conditions in agricultural systems [77,78].

The accumulation of heat at 12:00 and 18:00 in the traditional and integrated systems, as well as at 18:00 and 00:00 in the silvopastoral system, is associated with the highest rectal temperature index observed in cattle during the period of the experiment. This relationship between the accumulation of heat and the increase in rectal temperature reflects the direct influence of the thermal conditions of the environment in the animals, a fact evidenced in the analysis using infrared thermography, which showed higher temperature indices in the traditional system. Rectal temperature is an important indicator of the physiological response of cattle to heat stress, and its elevation can be attributed to the body’s effort to dissipate excessive heat to maintain homeostasis [79,80,81,82,83,84,85,86].

During peak solar hours, such as 12:00 and 18:00, solar radiation falls directly on the animals, resulting in a significant increase in ambient temperature. In traditional and integrated systems, where there is not adequate shading, cattle are exposed to intense heat, which can lead to an excessive accumulation of heat in their bodies. Lack of shade can also limit the ability of cattle to shelter and protect themselves from the heat, aggravating heat stress and contributing to increased RT [87,88].

In the silvopastoral system, the greater accumulation of heat observed at 18:00 p.m. and 00:00 p.m. can be explained by the effect of solar radiation accumulated during the day. Trees provide shade throughout the day, which can reduce ambient temperatures compared to traditional and integrated systems. However, as solar radiation decreases after sunset, the heat stored on tree and soil surfaces begins to be released, causing the ambient temperature to rise at these times. This phenomenon is known as the “heat island effect”, which can be observed in shaded areas at night [51,89,90].

The results of the THI evaluation indicated no thermal stress at 7:00 a.m., with values below 72, which suggests that this time is more favorable in terms of thermal conditions for cattle. However, at 6:00 a.m., 8:00 a.m., 9:00 a.m., and from 7:00 p.m. onwards, lower THI indices were observed, characterizing a mild stress in the animals in these periods. This result may be related to the beginning of the day, when the ambient temperature has not yet reached its peak, and to the afternoon and night, when the temperature begins to decrease. These times of day can offer some thermal relief for the animals, especially if they are in an environment with adequate shading, as thermal comfort affects the performance of beef cattle [91].

On the other hand, the hours from 10:00 a.m. to 6:00 p.m. showed higher THI indices, indicating moderate thermal stress. These are the periods of greater solar intensity when environmental temperatures are higher and cattle are more susceptible to thermal stress. During these hours, direct exposure to solar radiation can lead to excessive accumulation of heat in animals, increasing body temperature and making thermoregulation difficult [32,92].

The BGHI showed that the hours of 6:00 a.m., 7:00 a.m., and 8:00 a.m. are more comfortable for cattle, with a low risk of thermal stress. This may be associated with nocturnal cooling and lower solar intensity during this period. In addition, from 11:00 p.m. to 6:00 a.m., the BGHI indicated comfort, reflecting the drop in temperatures during the night and early morning [39].

However, between 1:00 p.m. and 10:00 p.m., the BGHI presented moderate thermal stress (75–78—warning on a scale of ≤74: situation of thermal comfort; 75–78: warning; 79–84: danger; and ≥85: emergency) [93]. This time range corresponds to the hottest moments of the day, when cattle are more exposed to direct solar radiation and the accumulation of heat in the environment. The lack of adequate shade and poor air circulation during this period can contribute to the thermal stress of the animals, impairing their well-being and performance [46,50,94,95].

The ESI corroborated the BGHI data, indicating moderate thermal stress at 1:00 p.m. and mild stress from 7:00 p.m. This coherence between the indices reinforces the importance of these times in the occurrence of thermal stress in animal production systems. The afternoon period presented a moderate risk of stress, probably due to the accumulation of heat in the environment during the day and the absence of efficient cooling mechanisms for the animals [39].

The ETI signaled a state of caution (≥27 to ≥32) at 7 a.m., which suggests that at this time, cattle need attention regarding the thermal conditions of the environment. The other times showed no problems (18 to <27), which may be related to the early morning and the night shift, when temperatures tend to be milder. These results reinforce the importance of monitoring thermal conditions throughout the day to ensure the comfort and well-being of cattle, especially in environments with greater exposure to solar radiation and without adequate protection against heat. Corroborating with the results of this research, Campos et al. [6], in the rural area of Bela Vista de Goiás, showed ETI averages equal to 26 for both treatments (sun exposure and shade), with ETI values greater than 27 being considered as a situation of discomfort.

With regard to the BTCI, all values were greater than 2.0, indicating difficulties in the animals’ adaptability to the environment. This result reinforces the importance of providing adequate thermal comfort conditions for cattle, especially in production systems with less shading, as in the traditional system. The lack of thermal comfort can lead to a series of negative impacts on the health and performance of animals, affecting their growth, production, and reproduction [32,39,96].

On the other hand, the BTCI showed a significant difference between the production systems, being higher in the traditional system compared to the silvopastoral and integrated systems. This indicates that cattle in the traditional system had greater difficulty in adapting to the environment in relation to the other systems, as values close to two are considered to provide greater animal comfort; that is, the cattle would be presenting RT and RR considered ideal [27]. This difference can be attributed to the lack of adequate shading in the traditional system, which exposes animals directly to solar radiation, increasing thermal stress and compromising their comfort and well-being [41,97].

The morning shift had the highest index of Iberian HTI, approaching 100 (the closer to 100, the more adapted the animal [98]), indicating a greater tolerance to heat for part of the cattle during this period. This result can be explained by the drop in temperatures during the night and the cooling at night, providing a milder environment for the animals in the early hours of the day [39]. Similar results were described by Marins et al. [4] in an experiment on a farm located in the tropical savanna from February to November, which obtained higher Iberian HTI results during the morning period (104.9 to 116.0); in the afternoon, they were 85.4 to 107.0 during all months.

Among the study limitations are the acquisition distance of the thermograms or the potential need to use an alternative lens and the absence of repeated measurements over a specific time interval.

## 5. Conclusions

The results of the thermographic images indicated that the silvopastoral system, with shading by chestnut trees and an ample vegetative area, provided more favorable thermal conditions compared to the traditional and integrated systems. Physiological analyses showed that the cattle maintained rectal temperatures within normal ranges in all systems. The respiratory rate was higher in the traditional system, indicating possible thermal stress. The thermal comfort indices pointed to moderate stress conditions at times of higher solar intensity. Considering the thermal stress indices evaluated, the silvopastoral system proved to be more suitable for the well-being of the animals, with lower thermal stress levels throughout most of the day. On the other hand, the traditional system showed higher levels of thermal stress, especially during hours of peak solar intensity. The integrated system occupied an intermediate position between the other two systems, with thermal stress values varying depending on the time of day.

It is important to emphasize that studies involving a larger number of animals, conducted across different seasons of the year, and especially with a greater number of repetitions, will provide a more definitive insight and confirmation as to whether a silvopastoral system, with shading from chestnut trees and ample vegetative cover, indeed offers more favorable thermal conditions compared to traditional and integrated systems.

## Figures and Tables

**Table 1:** Descriptive analysis of the results of the analysis of thermal data of the targets analyzed in the technological reference unit (TRU) in the different production systems in the eastern Amazon.

Variables	Systems		
	Silvopastoral	Traditional	Integrated
Sample size	10	10	10
Minimum	23.90	34.10	31.00
Maximum	30.30	36.70	40.40
Total Amplitude	6.40	2.60	9.40
Median	29.45	36.30	32.15
First Quartile (25%)	29.0250	36.1500	31.5500
Third Quartile (75%)	29.8750	36.3750	37.3000
Interquartile deviation	0.8500	0.2250	5.7500
Arithmetic average	28.98 a	35.93 b	34.11 b
Variance	3.40	0.96	15.52
Standard deviation	1.84	0.98	3.94
Standard Error	0.58	0.31	1.25
Coefficient of variation	6.36%	2.73%	11.55%
Asymmetry	−2.79	−1.62	1.02
Kurtosis	8.32	1.12	−1.11

Note: Arithmetic average: a,b different letters in the line indicate statistical differences (*p* < 0.05). Description of the systems: Silvopastoral—with shade and no access to the bathing area. In this system, the animals were submitted to pasture with the presence of trees and other elements that could provide shade; they did not have access to the bathing area. Traditional—no shade and no access to the bathing area. In this system, the animals were subjected to pasture without the presence of trees or other elements that could provide shade; they did not have access to the bathing area. Integrated—with access to shade and bathing area. In this system, the animals were subjected to pasture with trees and other elements that can provide shade, as well as access to the bathing area.

**Table 2:** Surface temperature indices according to the anatomical region of the body and the systems studied in the eastern Amazon.

Variables	Number of Data Points	Silvopastoral	Traditional	Integrated	Average	Standard Deviation
Head	2	35.05	36.7	34.9	35.05	0.815475322
Armpit	2	35.6	36.25	36.1	36.1	0.277888867
Flank	2	38.15	35.95	36.35	36.35	0.956846673
Rump	2	36.55	36.4	34.45	36.4	0.956556323
Average	2	35.6	36.25	36.1	36.1	0.277888867
Standard deviation	-	1.175996918	0.270416346	0.796084166	0.546008242	0.279560169

Note: Description of systems: Silvopastoral—with shade and no access to the bathing area. In this system, the animals were submitted to pasture with the presence of trees and other elements that could provide shade; they did not have access to the bathing area. Traditional—no shade and no access to the bathing area. In this system, the animals were subjected to pasture without the presence of trees or other elements that can provide shade; they did not have access to the bathing area. Integrated—with access to shade and bathing area. In this system, the animals were subjected to pasture with trees and other elements that can provide shade, as well as access to the bathing area.

**Table 3:** Indices used to evaluate the heat stress of cattle raised in silvopastoral (A), traditional (B), and integrated (C) systems from 6 a.m. to 6 a.m. during the experimental period in the eastern Amazon.

Time	THI	BGHI	ESI	ETI
06:00	72.716	70.426	23.08268	26.82964
07:00	69.62	68.43	20.85035	27.14537
08:00	73.996	71.256	23.95235	26.70116
09:00	76.832	74.512	26.36654	26.48436
10:00	81.35	74.82	28.16738	25.89942
11:00	81.438	73.478	27.7384	25.81436
12:00	81.978	72.828	27.72533	25.70745
13:00	82.818	74.308	28.51822	25.67495
14:00	84.336	75.876	29.61209	25.5546
15:00	83.11	76.25	29.33885	25.73756
16:00	83.686	76.826	29.33885	25.73756
17:00	83.29	76.43	29.63687	25.73874
18:00	80.546	75.546	28.18279	26.04266
19:00	77.646	74.646	26.74612	26.38379
20:00	77.498	75.308	26.96888	26.43312
21:00	76.84	74.81	26.48708	26.49706
22:00	76.976	74.796	26.48126	26.48495
23:00	74.036	72.666	24.4403	26.76465
00:00	73.908	72.878	24.46934	26.78917
01:00	73.9604	70.1604	23.57642	26.65066
02:00	73.7248	69.0548	23.13398	26.62408
03:00	73.804	68.844	22.91858	26.6229
04:00	75.2396	70.7796	24.34658	26.51007
05:00	72.5236	69.3436	22.65038	26.80276

Note: THI = temperature and humidity index, indicating <72 (green color); >72 to 78 (yellow color); and 79 to 88 (orange color) [31,32]. BGHI = black globe temperature and humidity index, indicating >72 comfort situation (green color) and 74 to 78 warning (yellow color) [26]. ESI = environmental stress index, indicating <25 low risk (green color) and ≤25 to ≤33 moderate to high risk (yellow color) [28]. ETI = equivalent temperature index, indicating no problems from 18 to <27 (green color) and caution ≥27 to ≥32 (yellow color) [29].

**Table 4:** Iberian Heat Tolerance Index (Iberian HTI) and Benezra Thermal Comfort Index (BTCI) during the experimental period in the eastern Amazon.

Times	Iberian HTI	BTCI
06H	87.34	2.330651
12H	82.54	2.418684
18H	79.24	2.510365
00H	84.94	2.342784

## Data Availability

The data presented in this study are available upon reasonable request from the corresponding author.
